# Supplementary material for: Amelioration of Hepatic Steatosis in Mice through Bacteroides uniformis CBA7346-Mediated Regulation of High-Fat Diet-Induced Insulin Resistance and Lipogenesis
Source: Nutrients. 2021 Aug 27;13(9):2989. doi: 10.3390/nu13092989 (PMC8471872; doi:10.3390/nu13092989)
Supplement: Supplementary file 1 [file nutrients-13-02989-s001.zip › B.uniformis_supplementary data_Nutrients.pptx]

## Slide 1
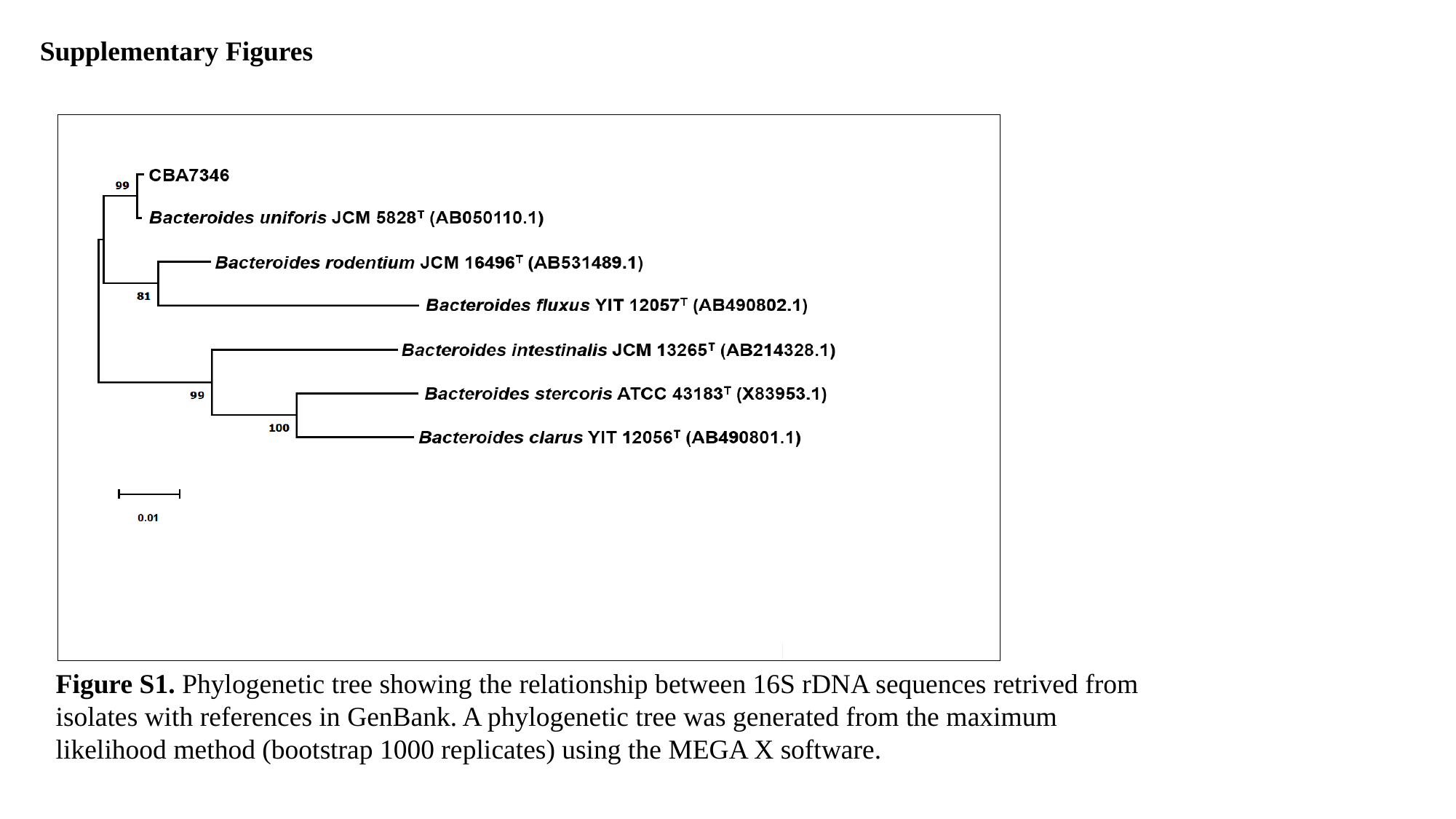

Supplementary Figures
Figure S1. Phylogenetic tree showing the relationship between 16S rDNA sequences retrived from isolates with references in GenBank. A phylogenetic tree was generated from the maximum likelihood method (bootstrap 1000 replicates) using the MEGA X software.
